# Predictors of Incident and Persistent Neck/Shoulder Pain in Iranian Workers: A Cohort Study

**DOI:** 10.1371/journal.pone.0057544

**Published:** 2013-02-28

**Authors:** Farideh Sadeghian, Mehdi Raei, Georgia Ntani, David Coggon

**Affiliations:** 1 Department of Occupational Health, Faculty of Health, Shahroud University of Medical Sciences, Shahroud, Iran; 2 Department of Biostatistics, School of Medicine, Qom University of Medical Sciences, Qom, Iran; 3 Medical Research Council Lifecourse Epidemiology Unit, University of Southampton, Southampton, United Kingdom; University of South Australia, Australia

## Abstract

**Background:**

Pain in the neck and shoulder has been linked with various psychosocial risk factors, as well as with occupational physical activities. However, most studies to date have been cross-sectional, making it difficult to exclude reverse causation. Moreover, they have been carried out largely in northern Europe, and the relationship to psychosocial factors might be different in other cultural environments.

**Methods:**

To explore causes of neck/shoulder pain, we carried out a longitudinal study in Iranian nurses and office workers. Participants (n  = 383) completed a baseline questionnaire about neck/shoulder pain in the past month and possible risk factors, and were again asked about pain 12 months later. Associations with pain at follow-up were explored by Poisson regression and summarised by prevalence rate ratios (PRRs).

**Results:**

After adjustment for other risk factors, new pain at follow-up was more frequent in office workers than nurses (PRR 1.9, 95%CI 1.3–2.8), among those with worst mental health (PRR 1.8, 95%CI 1.0–3.0), in those who reported incentives from piecework or bonuses (PRR1.4, 95%CI 1.0–2.0), and in those reporting job dissatisfaction (PRR 1.5, 95%CI 1.0–2.1). The strongest predictor of pain persistence was somatising tendency.

**Conclusions:**

Our findings are consistent with a hazard of neck/shoulder pain from prolonged use of computer keyboards, although it is possible that the association is modified by health beliefs and expectations. They also indicate that the association of low mood with neck/shoulder pain extends to non-European populations, and is not entirely attributable to reverse causation. Psychosocial aspects of work appeared to have relatively weak impact.

## Introduction

Pain in the neck and shoulder is an important cause of morbidity and incapacity for work in many countries, and often is attributed to physical activities in the workplace such as sustained abnormal posture, forceful repetitive tasks with the arms, work with the hands above shoulder height, and prolonged use of computer keyboards [1]–[3]. It has also been linked with various psychosocial risk factors [1], but most studies to date have been cross-sectional, making it difficult to exclude reverse causation (e.g. that the occurrence of pain leads to lower mood and perceived dissatisfaction with work). Moreover, they have been carried out largely in northern Europe, and the relationship to psychosocial factors might be different in other cultural environments.

One cross-sectional study of hospital nurses in Shiraz, Iran, found no significant association of neck or shoulder pain with perceived psychological demands [4], while another more recent survey of operating room nurses in the same city suggested a relation to shift work and (for shoulder pain) conflicting demands [5]. However, we are not aware of any previous longitudinal studies of neck/shoulder pain in Iranian workers.

As part of the international CUPID (Cultural and Psychosocial Influences on Disability) study [6], we analysed the longitudinal predictors of incident and persistent neck/shoulder pain in samples of nurses and office workers from Shahroud in north-eastern Iran.

## Methods

The study sample comprised nurses from three university hospitals and office workers who regularly used computers from the same hospitals and from four universities, all in Shahroud. We attempted to recruit all nurses and computer-using office workers aged 20–59 years, who were employed at the participating hospitals and universities, and had been working in their current job for >12 months. Potential participants were identified through a nominated manager at each organisation, and asked to complete a self-administered baseline questionnaire. The nominated managers did not carry out any selection among those eligible for study.

The questionnaire was a Farsi translation of the English language CUPID questionnaire, checked by independent back-translation, and piloted in workers not included in the current study [6]. Among other things, it asked about demographic characteristics, physical activities at work, psychosocial aspects of work, mental health, somatising tendency, and experience of pain lasting a day or longer during the past month at various anatomical sites (depicted in diagrams) including the neck, right shoulder and left shoulder.

The section about activities at work included inquiry as to whether an average working day entailed: a) use of a keyboard or typewriter for more than four hours in total; b) other tasks involving repeated movements of the wrist or fingers for more than four hours in total; c) work with hands above shoulder height for >1 hour; and d) lifting weights of 25 Kg or more by hand. The questions on psychosocial aspects of work covered: incentives from piecework or bonuses; time pressure; lack of choice in what work is done, how and when; lack of support from colleagues or supervisor/manager; job dissatisfaction; and perceived job insecurity if off work for three months with illness.

Mental health was scored using the relevant domain of the SF-36 questionnaire [7], and classified to three levels (corresponding to approximate thirds of the distribution in the full CUPID study). Somatising tendency was assessed using elements of the Brief Symptom Inventory [8], and graded according to the number of somatic symptoms from a total of five (faintness or dizziness, pains in the heart or chest, nausea or upset stomach, trouble getting breath, hot or cold spells) that had been at least moderately distressing during the past week. Neck/shoulder pain was defined as pain in the neck and/or either shoulder.

The questionnaire was accompanied by a brief written introduction explaining that the study was a survey of musculoskeletal disorders among workers. In addition, the lead researcher briefed the nominated managers and the heads of departments in which participants worked about the content of the questionnaire. Questionnaires were anonymised using a serial number, and returned to the research team via the nominated manager in a sealed envelope.

After an interval of 12 months, participants were asked to complete a second shorter questionnaire, which again asked about musculoskeletal pain in the past month, using identical questions.

Analysis was carried out with Stata Version 11.1 software. Associations between baseline risk factors and neck/shoulder pain at follow-up were assessed by Poisson regression with robust confidence intervals [9]. We first focused on subjects who were free from pain in the past month at baseline, and examined risk factors for the presence of new pain (incident pain) at follow-up. We then carried out a second analysis restricted to subjects who reported pain in the past month at baseline, in which we explored risk factors for the continuing presence of pain at follow-up (persistent pain). Associations were summarised by prevalence rate ratios (PRRs) and associated 95% confidence intervals (95%CIs).

Ethical approval for the study was provided by the Research Committee of Shahroud University of Medical Sciences. Information about the study was given to potential participants when they were first contacted, and consent to the baseline survey was deemed to be implicit in the return of a completed questionnaire. Written consent to receive the follow-up questionnaire was obtained as part of the baseline survey.

## Results

Baseline questionnaires were answered by 246 nurses and 182 office workers (response rates of 94% and 88% respectively among those invited to take part), and analysis was based on the 218 nurses (89%) and 165 office workers (91%) who completed follow-up. There was no major variation in the response rate at follow-up according to baseline characteristics of interest, including report of neck/shoulder pain (Table 1).

**Table 1:** Response rates at follow-up according to characteristics assessed at baseline.

Characteristic	Completed baseline questionnaire		Completed follow-up questionnaire		Response rate (%)	
	Nurses	Office workers	Nurses	Office workers	Nurses	Office workers
**Sex**						
Male	45	64	41	56	91.1	87.5
Female	201	118	177	109	88.1	92.4
**Age (years)**						
20–39	80	90	67	80	83.8	88.9
30–39	115	63	105	60	91.3	95.2
40–59	51	29	46	25	90.2	86.2
**Occupational activities**						
Work with hands above shoulderheight >1 hour	106	73	95	66	89.6	90.4
Lifting weights of ≥25 kg by hand	61	13	52	11	85.2	84.6
**Psychosocial aspects of work**						
Incentives	71	54	63	48	88.7	88.9
Time pressure	222	135	196	124	88.3	91.9
Lack of choice	61	34	54	32	88.5	94.1
Lack of support	58	49	50	40	86.2	81.6
Job dissatisfaction	72	48	67	42	93.1	87.5
Perceived job insecurity	135	121	123	109	91.1	90.1
**Mental health**						
Good	62	44	51	41	82.3	93.2
Intermediate	89	54	84	46	94.4	85.2
Poor	95	84	83	78	87.4	92.9
**Number of distressing somatic symptoms in past week**						
0	106	85	94	79	88.7	92.9
1	51	44	48	36	94.1	81.8
≥2	89	53	76	50	85.4	94.3
**Neck/shoulder pain in past month at baseline**						
No	169	105	150	95	88.8	90.5
Yes	77	77	68	70	88.3	90.9
**All subjects**	246	182	218	165	88.6	90.7

Among the subjects who completed follow-up, almost all of the office workers (98%) but relatively few of the nurses (10%) reported using a computer keyboard for more than four hours per day. However, most nurses said that they carried out other repetitive tasks with their wrists or fingers for more than four hours per day, and 24% that they routinely performed heavy manual lifting. At baseline, 138 participants (31% of nurses and 42% of office workers) reported neck/shoulder pain in the past month. Of these, 89 (64%) still had pain at follow-up, while among the 245 subjects who had been free from neck/shoulder pain at baseline, 79 (32%) had developed the symptom at follow-up.

Table 2 shows associations of risk factors at baseline with the subsequent incidence of new neck/shoulder pain among those who were free from the symptom at baseline. After adjustment for sex, age, and all of the other risk factors under consideration, incident pain was more frequent in office workers than nurses (PRR 1.9, 95%CI 1.3–2.8), in those who reported incentives from piecework or bonuses (PRR 1.4, 95%CI 1.0–2.0), in those reporting job dissatisfaction (PRR 1.5, 95%CI 1.0–2.1), and in those with poorest mental health (PRR 1.8, 95%CI 1.0–3.0). In an analysis that adjusted only for sex, age and occupation, there was also an association with work with the hands above shoulder height (PRR 1.4, 95%CI 1.0–1.9), but it was attenuated after adjustment for other risk factors.

**Table 2:** Associations of risk factors at baseline with incidence of new neck/shoulder pain at follow-up.

Risk factor	Number pain-free at baseline	Number (%) with pain at follow-up	Partially adjusted^a^		Fully adjusted^b^	
			PRR	(95%CI)	PRR	(95%CI)
**Occupation**						
Nurses	150	38 (25%)	1		1	
Office workers	95	41 (43%)	1.8	(1.2–2.5)	1.9	(1.3–2.8)
**Occupational activities**						
Work with hands above shoulderheight >1 hour	92	36 (39%)	1.4	(1.0–1.9)	1.2	(0.9–1.8)
Lifting weights of ≥25 kg by hand	45	16 (36%)	1.3	(0.8–2.1)	1.3	(0.8–2.1)
**Psychosocial aspects of work**						
Incentives	67	28 (42%)	1.4	(1.0–2.0)	1.4	(1.0–2.0)
Time pressure	206	64 (31%)	1.0	(0.6–1.6)	1.0	(0.6–1.7)
Lack of choice	55	17 (31%)	1.0	(0.6–1.5)	0.9	(0.6–1.4)
Lack of support	44	12 (27%)	0.9	(0.5–1.4)	0.8	(0.5–1.3)
Job dissatisfaction	66	26 (39%)	1.4	(1.0–2.1)	1.5	(1.0–2.1)
Perceived job insecurity	141	48 (34%)	1.1	(0.8–1.6)	1.0	(0.7–1.4)
**Mental health**						
Good	68	16 (24%)	1		1	
Intermediate	86	25 (29%)	1.2	(0.7–2.2)	1.3	(0.8–2.3)
Poor	91	38 (42%)	1.7	(1.0–2.8)	1.8	(1.0–3.0)
**Number of distressing somatic symptoms in past week**						
0	129	39 (30%)	1		1	
1	45	14 (31%)	1.1	(0.7–1.8)	0.9	(0.6–1.5)
≥2	71	26 (37%)	1.3	(0.9–1.9)	1.2	(0.8–1.8)

Analysis was restricted to the 245 subjects who were free from neck/shoulder pain at baseline.

a Adjusted for sex, age and occupation.

b Adjusted for all of the risk factors in the table.

Table 3 summarises associations with the persistence of neck/shoulder pain at follow-up among subjects who reported having suffered from such pain in the month before baseline. The strongest predictor of pain persistence was somatising tendency (PRR 1.4, 95%CI 1.1–1.9 for report of ≥2 v 0 distressing somatic symptoms in past week with adjustment for sex, age and occupation). However, this association ceased to be statistically significant after adjustment also for other risk factors.

**Table 3:** Associations of risk factors at baseline with persistence of neck/shoulder pain at follow-up.

Risk factor	Number withpain at baseline	Number (%) withpain at follow-up	Partially adjusted^a^		Fully adjusted^b^	
			PRR	(95%CI)	PRR	(95%CI)
**Occupation**						
Nurses	68	41 (60%)	1		1	
Office workers	70	48 (69%)	1.2	(0.9–1.5)	1.2	(0.9–1.5)
**Occupational activities**						
Work with hands above shoulderheight >1 hour	69	44 (64%)	1.0	(0.8–1.3)	1.0	(0.8–1.2)
Lifting weights of ≥25 kg by hand	18	11 (61%)	1.0	(0.7–1.5)	1.1	(0.8–1.7)
**Psychosocial aspects of work**						
Incentives	44	23 (52%)	0.8	(0.6–1.0)	0.7	(0.5–1.0)
Time pressure	114	76 (67%)	1.3	(0.9–1.8)	1.3	(0.9–2.0)
Lack of choice	31	19 (61%)	0.9	(0.7–1.3)	0.9	(0.7–1.3)
Lack of support	46	28 (61%)	0.9	(0.7–1.2)	0.9	(0.7–1.1)
Job dissatisfaction	43	27 (63%)	0.9	(0.7–1.2)	0.9	(0.7–1.1)
Perceived job insecurity	91	61 (67%)	1.2	(0.9–1.5)	1.2	(0.9–1.6)
**Mental health**						
Good	24	15 (63%)	1		1	
Intermediate	44	23 (52%)	0.8	(0.5–1.2)	0.9	(0.6–1.3)
Poor	70	51 (73%)	1.2	(0.8–1.6)	1.1	(0.8–1.6)
**Number of distressing somatic symptoms in past week**						
0	44	24 (55%)	1		1	
1	39	23 (59%)	1.1	(0.8–1.6)	1.1	(0.7–1.6)
≥2	55	42 (76%)	1.4	(1.1–1.9)	1.3	(0.9–1.8)

Analysis was restricted to the 138 subjects who reported neck/shoulder pain in the past month at baseline.

a Adjusted for sex, age and occupation.

b Adjusted for all of the risk factors in the table.

## Discussion

In this study, both the baseline prevalence and subsequent incidence of neck/shoulder pain were higher in office workers than in nurses, which would be compatible with a hazard from prolonged use of computer keyboards. Furthermore, the incidence of neck/shoulder pain was significantly predicted by low mood at baseline, indicating that the association of low mood with pain extends to non-European populations and does not arise simply because pain makes people miserable. Incident pain was also associated with job dissatisfaction and financial incentives from piecework and bonuses, but overall, the impact of psychosocial aspects of work was relatively weak.

The aim of the CUPID study was to recruit at least 200 participants from each of the occupational groups surveyed, which would give adequate statistical power for international comparisons [6]. To achieve this target in Iran, it was necessary to approach office workers from universities as well as from the hospitals at which the nurses worked. However, the methods by which the nurses and office workers were recruited were otherwise similar. In particular, the nominated mangers did not make any selection from among those eligible for study (e.g. on the basis of their history of musculoskeletal illness). Furthermore, response rates, both at baseline and at follow-up, were high, and the response at follow-up did not vary importantly according to whether neck/shoulder pain was present at baseline, or in relation to the risk factors of interest. Thus, it seems unlikely that our findings will have been subject to major selection bias.

The questionnaire that we employed was a Farsi translation of the CUPID questionnaire [6]. The components covering mental health and somatising tendency were taken from previously validated instruments [7], [8], while the questions on musculoskeletal pain were similar in style to the Nordic questionnaire [10], and those about occupational physical activities had been used successfully in earlier investigations [11]–[13]. Moreover, the accuracy of translation to Farsi was checked by independent back-translation. Nevertheless participants’ recall, especially of occupational activities, may not have been completely accurate. After stratification according to the presence of pain at baseline, any errors in answering the initial questionnaire are likely to have been non-differential in relation to subsequent symptoms, and thus will have tended to obscure associations between exposures and pain. However, it is possible that at follow-up, some subjects were more aware of pain, and therefore more likely to report it, if they carried out physical activities that stressed the shoulders or neck. If this occurred, it will have tended to inflate risk estimates for such activities.

Ideally, symptoms would have been ascertained repeatedly at intervals over follow-up, but this was not practical with the resource available. Therefore, follow-up was restricted to a single time-point. Nevertheless, the longitudinal design allowed stronger conclusions about directions of cause and effect than is possible from cross-sectional surveys. In particular, in the analysis of risk factors for incidence of new symptoms, poor mental health was ascertained at a time when subjects were free from neck-shoulder pain, and therefore it could not have been a consequence of such pain.

In our statistical analysis, we opted to use Poisson rather than logistic regression, because for a relatively frequent outcome such as neck/shoulder pain, the prevalence rate ratios which it estimates are more naturally interpretable than odds ratios. The validity of the method in such circumstances is well established [9].

The association of poor mental health with subsequent incidence of pain is consistent with findings from other longitudinal studies [14], [15]. It may be that people with lower mood are more aware of painful stimuli when they occur. Another possibility is that some individuals are generally more predisposed to report symptoms, whether psychological or physical. This would accord with the association that is commonly observed between musculoskeletal complaints and somatising tendency [16]–[19], although in our study neither the incidence nor persistence of neck-shoulder pain was strongly predicted by report of distress from common somatic symptoms.

After adjustment for other risk factors, incident neck/shoulder pain was more common in office workers than nurses, and this may have been related to their prolonged use of computer keyboards – our aim had been to enrol office workers who regularly used computers, and almost all confirmed that they used a keyboard for more than four hours in an average working day. A number of previous studies have linked computer work with neck/shoulder pain [3], [20], [21], although the finding has not been universal [3], and in a recent longitudinal study there was no relation to objectively measured duration of keyboard use [22] The heterogeneity of findings from previous studies might be explained if the postural stresses imposed by use of a computer keyboard cause transient aches and pains, but the transition to more persistent chronic symptoms is a psychologically mediated nocebo effect that depends importantly on prior health beliefs and expectations. This hypothesis will be tested in analysis of the full CUPID study.

We did not attempt to assess the relation of neck/shoulder pain to reported use of computer keyboards directly, because in our study sample, the exposure was strongly correlated with job category (98% of office workers v only 10% of nurses). Moreover, individual assessments of duration of occupational activity are unlikely to be completely accurate. Such errors may explain why the association of neck/shoulder pain with work that entailed prolonged elevation of the arms above shoulder height was weaker than in some previous studies [23], [24]. Substantial numbers of participants (43% of nurses and 40% of office workers) indicated that they held their arms above shoulder height for longer than an hour in an average working day, which seems unlikely to be correct.

Some previous studies have linked neck/shoulder pain also with psychosocial aspects of work such as high demands, poor support and low control [1], [25]. We found weak associations of incident pain with job dissatisfaction and financial incentives to productivity. In general, however, workplace psychosocial factors appeared to have little impact in our study. This accords with findings from a recent systematic review of risk factors for the development of non-specific neck pain in office workers [26].

Most of the associations that we observed were with the incidence rather than the persistence of neck/shoulder pain. This may be because there are genuine differences between the initiators of musculoskeletal; symptoms and the factors that cause them to persist. However, it could also be a chance observation. Again, it will be possible to explore the question in more depth when the full CUPID study is analysed.

### Conclusions

Given the high prevalence of neck/shoulder pain that we found among computer users, there is a case for better organisation of work stations and of work routines where this has potential to make the performance of tasks more comfortable and perhaps more efficient.
